# The Effect of 5-Hydroxytryptamine Receptor Antagonist in Preventing Pain/Limb Shrinkage Reaction Associated with Rocuronium Injection

**DOI:** 10.1155/2018/4128415

**Published:** 2018-07-12

**Authors:** Qixiong He, Chengmao Zhou, Yu Zhu

**Affiliations:** Zhaoqing Medical College, Zhaoqing 526020, China

## Abstract

**Objectives:**

To evaluate the effect and safety of 5-hydroxytryptamine (5-HT) receptor antagonist in alleviating the pain of patients under general anesthesia induced by rocuronium injection and preemptive analgesia.

**Methods:**

Meta-analysis was conducted with RevMan 5.1 software by electronically retrieving the databases of PubMed, Embase, the Cochrane Library, WanFang Data, and CNKI and collecting the published random control tests (RCTs) where 5-hydroxytryptamine receptor antagonist was used to alleviate the pain/limb shrinkage reaction associated with rocuronium injection.

**Results:**

Seven RTCs, including 556 patients, were included. The results of meta-analysis showed the following. (1) Compared to the control group, 5-HT receptor antagonist could prevent the total pain/limb shrinkage reaction associated with rocuronium injection [RR=0.62, 95% CI (0.54, 0.71), P<0.00001] and reduce the incidence rate of mild and moderate pain/limb shrinkage reaction associated with rocuronium injection [RR=0.46, 95% CI (0.33, 0.63), P<0.00001; RR=0.18, 95% CI (0.11, 0.31), P<0.000 01]. (2) Compared to the lidocaine group, the incidence rate was higher in preventing the pain/limb shrinkage reaction associated with rocuronium injection by 5-HT receptor antagonist, which was statistically significant [RR=1.33, 95% CI (1.05, 1.68), P=0.02].

**Conclusion:**

5-hydroxytryptamine receptor antagonist may be effective in preventing the pain/limb shrinkage reaction associated with rocuronium injection.

## 1. Introduction

Rocuronium is the time nondepolarizing muscle relaxant of the steroid with the quickest effect. It has been widely used in clinical applications without adverse cardiovascular response and release of histamine. [1, 2] However, it was clinically found that injection pain occurred in intravenous injection of rocuronium under induction of general anesthesia [3]. The main presentations were burning pain at different degrees on the injection sites from complaints of the patients. The sign after anesthesia was involuntary flexion-extension activity of wrists and elbows. The possibility of such induced pain was up to 50-80% [4], and it was 83-94% for children [5]. Currently, clinical studies show that the incidence rate of rocuronium injection pain can be reduced with the dilution method and intravenous pretreatment with lidocaine, ondansetron, tramadol, or fentanyl.

However, the conclusions have not been consistent among several studies on preventing the pain/limb shrinkage reaction associated with rocuronium injection by 5HT3 receptor antagonist over the years [6, 7]. Meta-analysis on this regard is not available at present. On this account, we gave a comprehensive evaluation to the effect of 5HT3 receptor antagonist in preventing the pain/limb shrinkage reaction associated with rocuronium injection with meta-analysis.

## 2. Data and Methods

### 2.1. Inclusion Criteria

#### 2.1.1. Study Design

This study was a random control test.

#### 2.1.2. Study Object

Object of study was patients under general anesthesia operation.

#### 2.1.3. Interventions

The test group was administered with 5-HT receptor antagonist to prevent the pain/limb shrinkage reaction associated with rocuronium injection. The control group used placebo.

#### 2.1.4. Outcome Measures

① The incidence rate of pain/limb shrinkage reaction: pain grading was as follows: 0 score: no pain; 1 score: mild pain; 2 scores: moderate pain; 3 scores: severe pain. ② Limb shrinkage movement: grading was as follows: Grade 1: no reaction; Grade 2: wrist joints movement; Grade 3: arm (elbow, shoulder) movement; Grade 4: full body reaction (including other body movements, cough, or suffocation).

### 2.2. Retrieval Strategy

Computer-based retrieval was conducted in PubMed, Embase, the Cochrane Library, WanFang Data, and CNKI databases. RCTs were searched in terms of the prevention of the pain/limb shrinkage reaction associated with rocuronium injection with different interventions, with the time scope from the establishment of these databases to May, 2017. Meanwhile, the documents included in the study were retraced to supplement and obtain relevant documents. The search words included random, injection pain, withdrawal, rocuronium, ondansetron, 5-HT3 receptor antagonists, granistetron, troposetron, and palonosetron.

### 2.3. The Evaluation of Bias Risks in Document Selection, Data Extraction, and Included Studies

The evaluation of bias risks in document selection, data extraction, and included studies was conducted by two researchers on their own. Any discrepancy was settled through discussion or submitted to a third researcher for judgment. The self-made data extraction form was used to extract data, and the main extractions included ① general information of the inclusion studies, including name of the first author, date of publishing, and country of test; ② basic features of the study object, including sample size, age, and ASA classification; ③ intervention details; ④ interested outcome measures and result measurement data. The methodological quality of random control test (RCT) was evaluated by the amended Jadad Scale, and the evaluation contents included ① random method; ② allocation concealment; ③ blind method (the implementer and the participant).

### 2.4. Statistical Method

RevMan 5.1 software was used for meta-analysis. The included studies were first tested for heterogeneity. The heterogeneity during the test was tested by chi-square (the test criterion was *α* =0.05). When the study results were not heterogeneous (*α*>0.05), the fixed effects model was used for meta-analysis. When the study results were heterogeneous, the cause for such heterogeneity was found, following which the random effects model was used for meta-analysis. The count variable was expressed in relative risk (RR) and its 95% CI. The continuous variable was expressed in weighted mean deviation (WMD) and its 95% CI. P was less than 0.05%, indicating that it was statistically significant. If the data from clinical control tests were unavailable for meta-analysis, they would only be qualitatively analyzed on a descriptive basis.

## 3. Results

### 3.1. Retrieval Results

**Figure 1:**
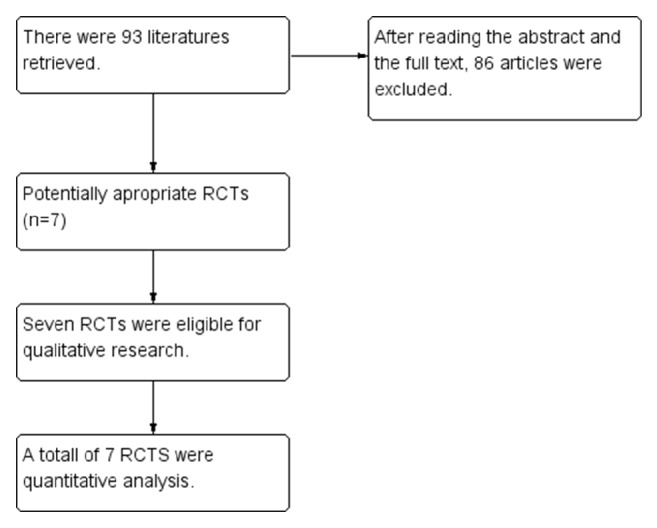
Flow diagram.

93 documents were initially searched according to the retrieval strategy above. The 93 documents were initially screened by reading the document titles and abstracts. Further reading was conducted for the second screening. After exclusion of 1 document for which the statistical data were unavailable [8–14], 7 documents were finally included (see Figure 1).

### 3.2. Basic Information of Included Studies and Evaluation of Methodological Quality

See Table 1.

### 3.3. Results of Meta-Analysis

**Figure 2:**
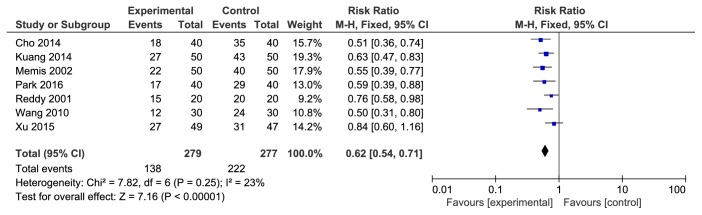
The pain/limb shrinkage reaction associated with rocuronium injection.

There were a total of 7 studies reporting that 5-HT receptor antagonist could prevent the pain/limb shrinkage reaction associated with rocuronium injection. The studies were statistically heterogeneous (P=0.25). The fixed effects model was used for meta-analysis. The results showed that the difference between both groups was statistically significant [RR=0.62, 95%CI (0.54, 0.71), P<0.00001]. The incidence rate of 5-HT3 receptor antagonist was lower (see Figure 2).

**Figure 3:**
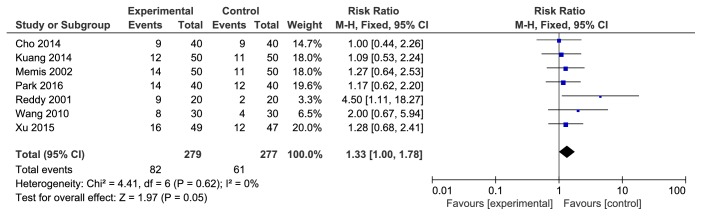
The incidence rate of mild pain/limb shrinkage reaction associated with rocuronium injection.

There were a total of 7 studies reporting the incidence rate of mild pain/limb shrinkage reaction associated with rocuronium injection. The studies were not statistically heterogeneous (P=0.62). The fixed effects model was used for meta-analysis. The results showed that the difference between the two groups was statistically significant [RR=1.33, 95%CI (1.00, 1.78), P=0.05] (see Figure 3).

**Figure 4:**
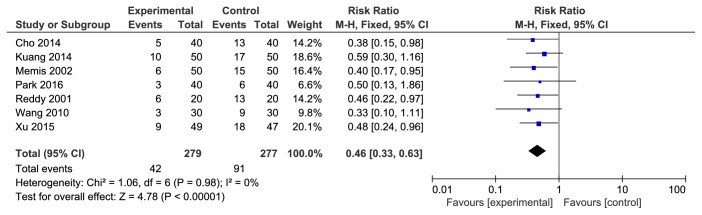
The incidence rate of moderate pain/limb shrinkage reaction associated with rocuronium injection.

There were a total of 7 studies reporting the incidence rate of moderate pain/limb shrinkage reaction associated with rocuronium injection. The studies were not statistically heterogeneous (P=0.98). The fixed effects model was used for meta-analysis. The results showed that the difference between the two groups was statistically significant [RR=0.46, 95% CI (0.33, 0.63), P<0.00001]. The incidence rate of 5-HT3 receptor antagonist was lower (see Figure 4).

**Figure 5:**
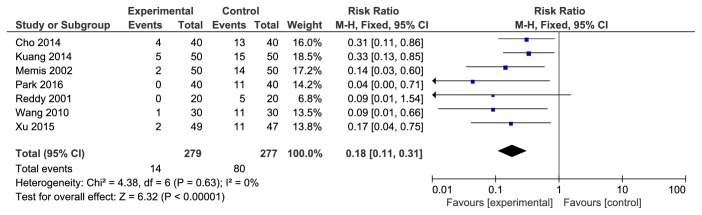
The incidence rate of severe pain/limb shrinkage reaction associated with rocuronium injection.

There were a total of 7 studies reporting the incidence rate of severe pain/limb shrinkage reaction associated with rocuronium injection. The studies were not statistically heterogeneous (P=0.63). The fixed effects model was used for meta-analysis. The results showed that the difference between the two groups was statistically significant [RR=0.18, 95% CI (0.11, 0.31), P<0.00001]. The incidence rate of 5-HT receptor antagonist was lower (see Figure 5).

**Figure 6:**
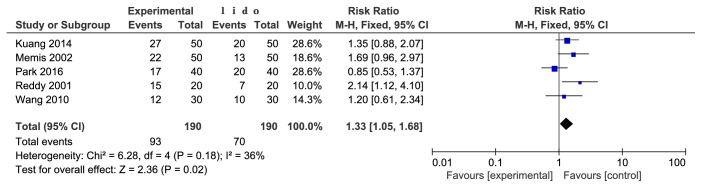
5-HT receptor antagonist and lidocaine prevented the pain/limb shrinkage reaction associated with rocuronium injection.

There were a total of 5 studies reporting that 5-HT receptor antagonist and lidocaine prevented the pain/limb shrinkage reaction associated with rocuronium injection. The studies were not statistically heterogeneous (P=0.18). The fixed effects model was used for meta-analysis. The results showed that the difference between the two groups was statistically significant [RR=1.33, 95% CI (1.05, 1.68), P=0.02]. The incidence rate of the lidocaine group was lower (see Figure 6).

### 3.4. Sensitivity Analysis and Funnel Plot Analysis

The funnel plot analysis showed symmetry, indicating that there was no publication bias. In the general meta-analysis, the studies were eliminated one by one for meta-analysis. The results were consistent with those before elimination, implying good stability.

## 4. Discussion

During anesthesia induction, intravenous injection of rocuronium may lead to intravenous injection pain to different degrees. The limb shrinkage reaction as a result of injection pain may cause incidents such as injury or dislocation of upper limbs of the patients as they are unconscious. The shift or dislocation of intravenous trocar caused by limb shrinkage reaction may result in interrupted anesthesia, intraoperative awareness, and seriously cardiovascular responses and even aspiration cough of the patients. The results of meta-analysis showed that 5-HT receptor antagonist may be effective in preventing the pain/limb shrinkage reaction associated with rocuronium injection.

The mechanism of rocuronium-induced injection pain remains unclear. Lockey and Coleman [6] held that local injecting pain caused by rocuronium was related to the stimulus of meta-acidic liquor to endangium. The study of Blunk et al. [15] showed that rocuronium molecules stimulated the mast cells and released a small amount of histamines and trypsin and stimulated the terminal of the nerve fibers after intravenously penetrating into the local injecting tissues. On the one hand, it led to axon reflex, thus resulting in vasodilatation and which was clinically represented as local erythema. On the other hand, the excitatory signal as a result of stimulus was transmitted to the centre, and the subject may feel local pains and itches. Currently, some studies [4, 16] have shown that, for adults and children, the dilution of rocuronium during intravenous injection can effectively reduce the incidence of injection pain. However, there are some disturbing factors in these studies, such as the fact that the injection speed of rocuronium may be one of the influencing factors; moreover, the effect of dilution of rocuronium has not been compared with that of other drug interventions.

Ondansetron is a commonly used drug for the prevention and treatment of nausea and vomiting clinically as a specific 5-HT3 receptor antagonist. It was found that its local anesthetic properties started from the prevention of propofol injection pain [17, 18]. The study of Ye et al. [19] found that 5-HT3 receptor antagonist could block the sodium channel of block the brain nerve cells of rats. It activated opioid receptors while inhibiting the release and uptake of norepinephrine to achieve analgesic effect.

Lidocaine is also the most commonly used drug for drug injection. According to Ye's research [19], a subcutaneous injection of ondansetron can produce about 15 times as much local anesthesia as lidocaine. The molecular structure of 5-ht3 receptor blockers is completely different from local anesthetics, but can produce similar local anesthesia. The limitations of this study included the following: ① limited documents to the inclusion criteria and relatively insufficient samples might lead to inadequate testing efficiency; ② only published documents were included; it could not be excluded that the study on negative results may not be included for analysis as it was not published; ③ in the included studies, the drugs used in the studies differed in the time of administration and the injection rate, both of which might have potential effect on the conclusion of comprehensive analysis. More high-quality studies with careful design are needed to reduce and decrease the effect of bias on the study results.

In conclusion, 5-HT receptor antagonist may be effective in preventing and reducing the occurrence of the pain/limb shrinkage reaction associated with rocuronium injection.

## Figures and Tables

**Table 1:** Characteristics and Jadad scores of the included studies in the meta-analysis.

Author (Published year)	Country	Headcount	Grouping (dose regimen)	Surgical setting	Application time
Reddy 2001	Singapore	60	Normal Saline Ondansetron 4 mg Lidocaine 50mg	elective orthopaedic and gastrointestinal procedures	One minute after the administration
Memis 2002	Turkey	250	Normal Saline Ondansetron 4 mg Lidocaine 30mg Tramadol 50 mg Fentanyl 0.1mg	Elective hysteroscopy or arthroscopy operations	After 20 s
Wang 2010	China	120	Normal Saline 2ml Lidocaine 20mg Ondansetron 4 mg Ondansetron 4 mg+Lidocaine 20mg	Laparoscopic surgery	One minute after the administration

Cho 2014	Korea	80	Normal Saline Ondansetron 4 mg	Elective surgery	Thirty seconds after the injection
Kuang 2014	China	200	Normal Saline palonosetron 0.25mg Lidocaine 40mg palonosetron 0.25mg+Lidocaine 40mg	Elective surgery	Thirty seconds after the injection
Xu 2015	China	200	Normal Saline 2ml troposetron 5 mg Dezocine 5mg Troposetron 5mg+Dezocine 5mg	Elective surgery	Two minute after the administration
Park 2016	Korea	120	Normal Saline 2ml Lidocaine 20mg palonosetron 0.075 mg	Laparoscopic surgery	Two minutes after the administration
